# Supplementary material for: A Breast Cancer Polygenic Risk Score Is Feasible for Risk Stratification in the Norwegian Population
Source: Cancers (Basel). 2023 Aug 16;15(16):4124. doi: 10.3390/cancers15164124 (PMC10452897; doi:10.3390/cancers15164124)
Supplement: Supplementary file 1 [file cancers-15-04124-s001.zip › cancers-2500179-supplementary.pdf]

SUPPLEMENTARY MATERIALS

A Breast Cancer Polygenic Risk Score is feasible for risk-stratification in the Norwegian population

Genome Data

The regional and statistical details about the dataset are given in Table S1. Statistics in this table are obtained by eliminating relatedness using KING [46], resulting in 6369 unrelated samples. The regional distribution of these unrelated samples and corresponding statistics are given in Table S1.

County	Abb	N	N*	Median sum of ROH	Mean IBD	Ne	Pop pr. km²	Pop	Ne/pop
**Østfold**	OF	388	200	5.51	4.22	396 000	56	221 386	1,79
**Akershus**	AK	1132	200	4.96	3.55	919 000	70	324 390	2,83
**Oslo**	OS	913	200	4.93	3.45	579 000	1127	481 548	1,20
**Hedmark**	HE	325	200	8.00	8.66	93 600	6	179 204	0,52
**Oppland**	OP	294	200	7.47	7.59	89 100	7	172 479	0,52
**Buskerud**	BU	388	200	5.59	5.63	204 000	14	198 852	1,03
**Vestfold**	VE	417	200	6.00	5.06	115 000	81	175 402	0,66
**Telemark**	TE	240	200	6.66	8.76	91 400	11	156 778	0,58
**Aust-Agder**	AA	158	152	8.19	9.77	118 000	9	80 839	1,46
**Vest-Agder**	VA	252	200	12.00	14.25	44 100	18	124 171	0,36
**Rogaland**	RO	225	200	8.41	15.20	27 600	31	268 682	0,10
**Hordaland**	HO	52	52	8.13	6.16	55 500	25	260 492	0,21
**Sogn og Fjordane**	SF	22	22	10.54	16.33	12 000	5	100 933	0,12
**Møre og Romsdal**	MR	187	187	7.84	9.87	270 000	15	223 709	1,21
**Sør-Trøndelag**	ST	1011	200	6.74	8.53	187 000	13	234 022	0,80
**Nord-Trøndelag**	NT	187	187	8.31	9.13	116 000	5	117 998	0,98

**Nordland**	NO	100	100	6.64	7.29	57 400	6	240 951	0,24
**Troms**	TR	54	54	8.84	11.50	25 600	5	136 805	0,19
**Finnmark**	FI	30	30	27.04	62.50	2600	2	39 757	0,07
**All**		6374	2984	6.82	4.18	-	12	3 888 305	-

Table S1. Summary statistics per region. N = the number of samples passing quality control. N* = the final number of random samples per county included in the analysis, with max 200. Mean ROH = mean sum of Runs-of-Homozygosity in cM. Mean IBD = Mean within-county IBD sharing in cM. Ne = estimate of effective population size at g = 5 ago. Pop. size and pop. per km2 = census population size in 1970.

In Figure S1, we have plotted the MAF comparison of our dataset with the UKB dataset and the Estonian dataset. As can be seen from this figure, Norwegian MAF values are in parallel with UKB and Estonian samples. This may also explain the results as coherent with (17).

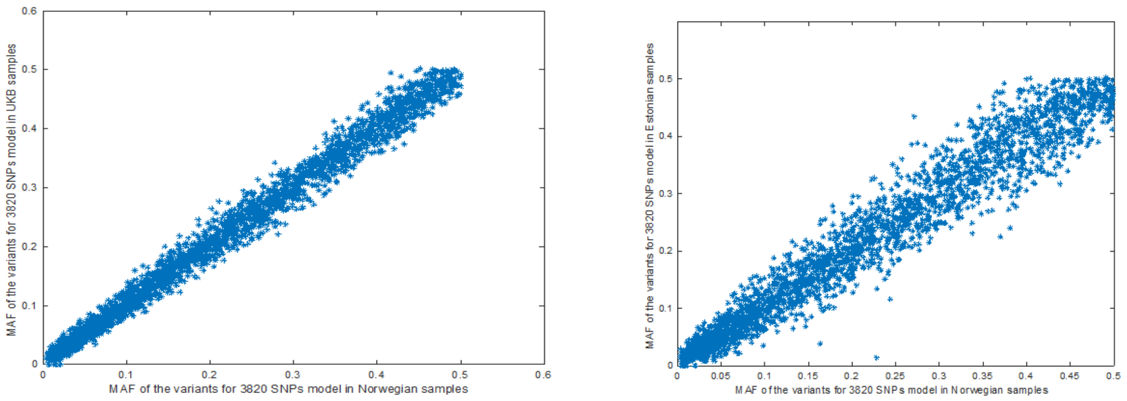

(A) Norwegian vs UKB

(B) Norwegian vs Estonian

Figure S1. MAF comparison of our dataset with the UKB dataset and the Estonian dataset for the 3820 SNPs model

Phenome Data

Our phenotype data includes 9,201 samples diagnosed with cancer. The distribution of the cancers in the dataset is given in Table S2. These samples are, aggregated in the 1980-1995 period, with survival data, information on all cancers occurring in these cases, time of last follow-up and also the time of diagnoses.

ICD9	DESCRIPTION	# of cases

**174**	**BREAST**	**1690**
**173**	**SKIN**	**3166**
**183**	**OVARY**	**734**
**153**	**COLON**	**666**
**154**	**RECTUM**	**279**
**162**	**LUNG**	**267**
**172**	**MELANOMA**	**391**
**185**	**PROSTATE**	**363**
**182**	**ENDOMETRIE**	**228**
**180**	**CERVIX**	**117**
**189**	**KIDNEY/URETER**	**139**
**193**	**THYROID**	**177**
	**Others**	**984**
	**TOTAL**	**9201**

Table S2. Distribution of cancers among 9201 samples.

The distribution of cases (BC diagnosis) and controls (samples without any cancer) concerning follow-up age is given in Figure S2. The mean value of the cases, controls and all samples with respect to follow-up are 47.95, 54.17 and 53.34, respectively. The incidence rate of BC in this dataset and Norway is plotted in Figure S2d. As can be seen in this figure, the peak of the incidence rate of this dataset is around the age of 40, while in the Norwegian population it is around 60. The main reason of this difference is that the samples collected in this dataset are chosen from the families with known cancer history.

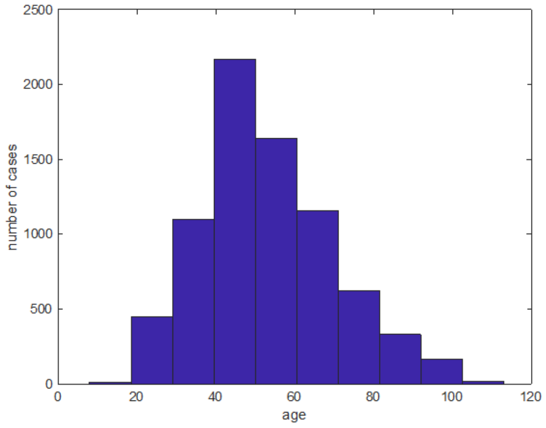

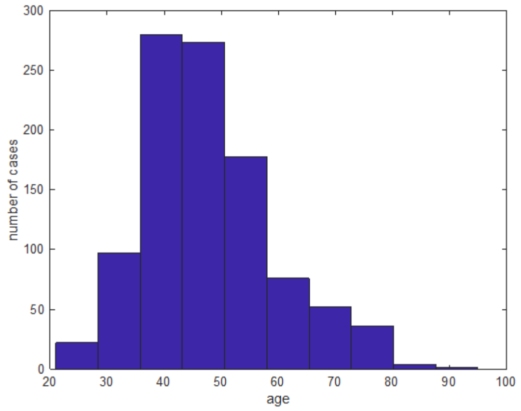

(a) Histogram of all samples with respect to age

(b) Histogram of cases with respect to age

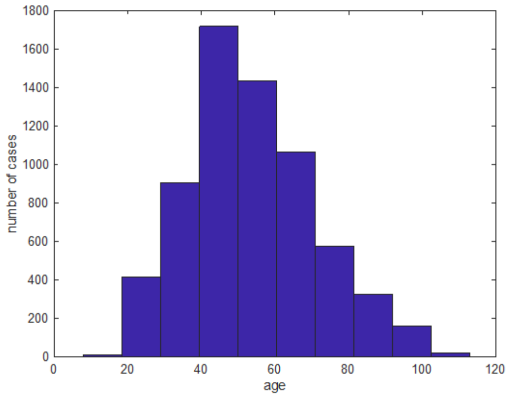

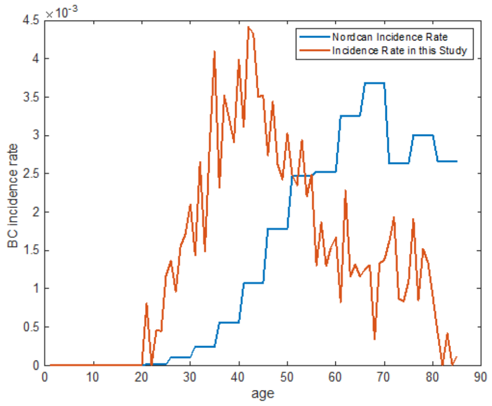

(c) Histogram of controls with respect to age

(d) Incidence rate obtained from our data vs NORDCAN

Figure S2. Histogram of the cases, controls and all samples with respect to follow-up age. The mean value of the cases, controls and all samples are 47.95, 54.17 and 53.34, respectively. In Figure A2d, the incidence rate of BC obtained from the histogram of our data and the incidence rate obtained from Nordcan are presented.

Effect of BRCA variants

Among these 9,201 samples with cancer, 3,223 had pathogenic germline variants in clinically actionable predisposition genes, including BRCA1 and BRCA2. In our resultant data for the analysis (1,053 samples for cases and 7,094 controls), 1166 of them have BRCA1 or BRCA2 variants (177 cases and 989 controls). We have excluded those 1166 samples, and have repeated the PRS analysis and calculated the corresponding AUC, OR and HR for 3820 SNPs model. As can be seen in Table S3, there is an increase in the performance of PRS (compared to Table 1) when we exclude BRCA1/2 carriers. This is expected and can also be observed in previous studies that BRCA1/2 carriers are less pronounced for PRS risk stratification compared noncarriers (16).

Metrics	SNPSET	Antegenes Pipeline (1kgp)	MoBa (HRC)	Norgene (Norwegian reference panel)
AUC (# of SNPs used)	3820 SNPs	0.630 (2706)	0.629 (2482)	0.638 (2698)
OR (se)	3820 SNPs	1.623 (0.035)	1.597 (0.035)	1.657 (0.035)
HR (%95 confidence interval)	3820 SNPs	1.510 (1.421-1.607)	1.491 (1.389-1.571)	1.542 (1.451-1.632)

Table S3 Performance of the dataset for 3820 SNPs when excluding 1166 samples that are BRCA1/2 carriers

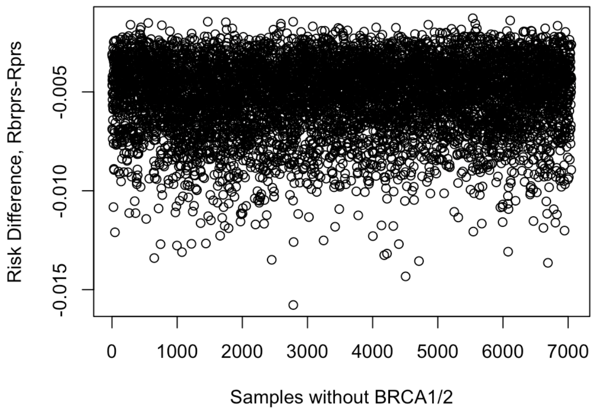

(a) For non-BRCA carriers

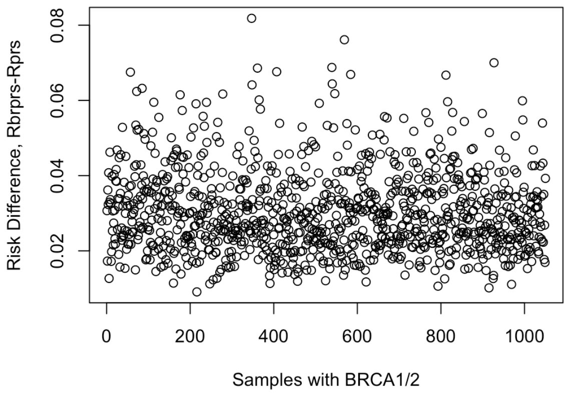

(b) For BRCA carriers

Figure S3. Cumulative risk differences of the samples when calculated with only PRS (Rprs) and with PRS and BRCA1/2 status (Rbrprs) . For non-BRCA carriers, there is a risk reduction with 0.5 percent mean while for BRCA1/2 carriers there is 3 percent increase when we added BRCA status as an additional risk factor.

Although there is a reduction in PRS performance when BRCA1/2 carriers are included, it is well known that there is an association of BRCA1/2 status with BC. We therefore performed an additional lifetime risk analysis, by combining PRS and the status of BRCA1/2, using iCare for the 3820 SNPs model. We compared the individuals' cumulative risk calculated using only PRS (Rprs), with the risks calculated using both PRS and BRCA1/2 status (Rbrprs). In Figure S3, we have plotted the risk differences, Rbrprs-Rprs. As observed from Figure S3a, in samples not being BRCA carriers, there is a slight decrease in the cumulative risk of having BC (mean:-0.005 and standard deviation:0.002). In other words, when we include BRCA1/2 status with PRS to asses the risk, there is a 0.5 percent risk reduction on the average for samples not being BRCA carriers. On the other hand, as can be seen

from Figure S3b, there is a an increase in cumulative risk ranging from 1 percent to 8 percent for BRCA1/2 carriers (mean:0.031, standard deviation:0.010).

Imputation with Norwegian Reference Panel

Imputation of untyped variation in the samples was conducted by impute5, using the 6369 phased dataset as query and the phased 1368 WGS dataset as reference. First, we converted the reference samples to imp5 format using the imp5Converter (v 1.1.5). Then we generated imputation chunks using imp5Chunker (v 1.1.5), generating coordinates of 5 Mbp chunks including 250 Kbp buffer regions. Finally, impute5 was run using the imp5 formatted WGS haplotypes as reference, and untyped variation in the dataset imputed, returning phases and dosages.

Imputation Pipelines

We have imputed the data in three different imputation pipelines. The details of these imputation pipelines have been presented in Table S4.1.

Imputation pipeline	Antegenes	MoBa	Norgene
Reference panel	1000G phase 3	HRC	Norwegian
Phasing	Eagle2	Shapeit2 [47]	Eagle2
Imputation	Beagle5 [48]	Impute4	Impute5 [49]
Notes	For details, see (17)	For details, see (28)	For details, see Imputation with Norwegian Reference Panel section

Table S4.1. Different imputation pipelines that are used to impute Norwegian genome data.

Using these imputation pipelines, the corresponding number of SNPs obtained for each PRS model is presented in Table S4.2**.**

SNPSET	Antgegenes Pipeline (1kgp)	MoBa (HRC)	Norgene (Norwegian reference panel)
77 SNPs (13)	76	75	72
313 SNPs (5)	262	239	287
2803 SNPs	2616	2553	2681
3820 SNPs (5)	3181	2893	3688

Table S4.2. Number of SNPs remaining for each model using different imputation pipelines.

Supplementary Risk Results

Since the calculated risks in Figure 2 in the main text visually look quite similar among different PRS models, another possible representation of Figure 2 can be done via numerical values in the tables as given below.

Age	1st percentile (%95 CI)	25th percentile (%95 CI)	50th percentile (%95 CI)	75th percentile (%95 CI)	90th percentile (%95 CI)	99th percentile (%95 CI)
30	0.0002 (0.0002,0.0002)	0.0003 (0.0004,0.0003)	0.0004 (0.0005,0.0004)	0.0006 (0.0006,0.0006)	0.0007 (0.0007,0.0008)	0.0011 (0.0010,0.0012)
40	0.0015 (0.0018,0.0013)	0.0029 (0.0030,0.0027)	0.0037 (0.0038,0.0037)	0.0049 (0.0048,0.0050)	0.0062 (0.0059,0.0064)	0.0092 (0.0084,0.0102)
50	0.0065 (0.0074,0.0056)	0.0119 (0.0127,0.0114)	0.0155 (0.0159,0.0153)	0.0205 (0.0201,0.0207)	0.0258 (0.0245,0.0268)	0.0383 (0.0347,0.0421)

60	0.0155 (0.0177,0.0135)	0.0285 (0.0303,0.0272)	0.0370 (0.0378,0.0364)	0.0487 (0.0476,0.0491)	0.0611 (0.0579,0.0633)	0.0898 (0.0816,0.0985)
70	0.0275 (0.0313,0.0241)	0.0503 (0.0533,0.0481)	0.0651 (0.0664,0.0641)	0.0853 (0.0834,0.0861)	0.1064 (0.1009,0.1102)	0.1545 (0.1408,0.1691)
80	0.0368 (0.0419,0.0323)	0.0671 (0.0711,0.0643)	0.0866 (0.0883,0.0853)	0.1130 (0.1104,0.1140)	0.1404 (0.1332,0.1453)	0.2019 (0.1844,0.2205)

Table S5.1 Absolute cumulative lifetime risks calculated using PRS values obtained from 3820 SNPs and HR (with its %95 confidence intervals)

Age	1st percentile (%95 CI)	25th percentile (%95 CI)	50th percentile (%95 CI)	75th percentile (%95 CI)	90th percentile (%95 CI)	99th percentile (%95 CI)
30	0.0002 (0.0002,0.0002)	0.0004 (0.0004,0.0003)	0.0005 (0.0005,0.0004)	0.0006 (0.0006,0.0006)	0.0007 (0.0007,0.0008)	0.0011 (0.0010,0.0012)
40	0.0016 (0.0018,0.0014)	0.0029 (0.0031,0.0028)	0.0038 (0.0038,0.0037)	0.0049 (0.0048,0.0049)	0.0061 (0.0058,0.0064)	0.0091 (0.0082,0.0101)
50	0.0067 (0.0076,0.0058)	0.0123 (0.0129,0.0116)	0.0157 (0.0160,0.0154)	0.0203 (0.0200,0.0206)	0.0253 (0.0242,0.0265)	0.0378 (0.0342,0.0416)
60	0.0160 (0.0183,0.0140)	0.0293 (0.0308,0.0278)	0.0373 (0.0380,0.0366)	0.0482 (0.0474,0.0489)	0.0599 (0.0572,0.0626)	0.0885 (0.0803,0.0974)
70	0.0284 (0.0323,0.0249)	0.0517 (0.0543,0.0491)	0.0656 (0.0667,0.0644)	0.0844 (0.0830,0.0857)	0.1044 (0.0997,0.1090)	0.1524 (0.1387,0.1672)
80	0.0380 (0.0432,0.0333)	0.0689 (0.0722,0.0655)	0.0872 (0.0886,0.0857)	0.1118 (0.1099,0.1136)	0.1377 (0.1317,0.1438)	0.1992 (0.1817,0.2181)

Table S5.2 Absolute cumulative lifetime risks calculated using PRS values obtained from 2803 SNPs and HR (with its %95 confidence intervals)

Age	1st percentile (%95 CI)	25th percentile (%95 CI)	50th percentile (%95 CI)	75th percentile (%95 CI)	90th percentile (%95 CI)	99th percentile (%95 CI)
30	0.0002 (0.0002,0.0002)	0.0004 (0.0004,0.0003)	0.0005 (0.0005,0.0004)	0.0006 (0.0006,0.0006)	0.0007 (0.0007,0.0008)	0.0011 (0.0010,0.0012)
40	0.0016 (0.0019,0.0014)	0.0030 (0.0031,0.0028)	0.0038 (0.0039,0.0037)	0.0048 (0.0047,0.0049)	0.0060 (0.0057,0.0063)	0.0089 (0.0081,0.0099)
50	0.0068 (0.0078,0.0060)	0.0124 (0.0130,0.0118)	0.0158 (0.0161,0.0155)	0.0201 (0.0198,0.0204)	0.0251 (0.0239,0.0262)	0.0371 (0.0335,0.0409)
60	0.0164 (0.0187,0.0143)	0.0296 (0.0311,0.0281)	0.0377 (0.0383,0.0370)	0.0477 (0.0469,0.0484)	0.0593 (0.0566,0.0620)	0.0870 (0.0788,0.0958)
70	0.0290 (0.0331,0.0254)	0.0522 (0.0548,0.0496)	0.0662 (0.0672,0.0650)	0.0836 (0.0821,0.0848)	0.1033 (0.0986,0.1079)	0.1499 (0.1361,0.1645)
80	0.0388 (0.0442,0.0340)	0.0695 (0.0729,0.0662)	0.0880 (0.0893,0.0865)	0.1107 (0.1088,0.1124)	0.1363 (0.1302,0.1423)	0.1960 (0.1784,0.2146)

Table S5.3 Absolute cumulative lifetime risks calculated using PRS values obtained from 313 SNPs and HR (with its %95 confidence intervals)

Age	1st percentile (%95 CI)	25th percentile (%95 CI)	50th percentile (%95 CI)	75th percentile (%95 CI)	90th percentile (%95 CI)	99th percentile (%95 CI)
30	0.0002 (0.0003,0.0002)	0.0004 (0.0004,0.0004)	0.0005 (0.0005,0.0005)	0.0006 (0.0006,0.0006)	0.0007 (0.0007,0.0007)	0.0010 (0.0009,0.0011)
40	0.0019 (0.0022,0.0017)	0.0031 (0.0032,0.0029)	0.0038 (0.0039,0.0038)	0.0047 (0.0046,0.0048)	0.0058 (0.0055,0.0061)	0.0083 (0.0075,0.0092)
50	0.0081 (0.0091,0.0071)	0.0129 (0.0136,0.0123)	0.0160 (0.0162,0.0157)	0.0197 (0.0194,0.0200)	0.0241 (0.0230,0.0252)	0.0344 (0.0310,0.0381)
60	0.0193 (0.0218,0.0170)	0.0308 (0.0323,0.0293)	0.0380 (0.0386,0.0374)	0.0468 (0.0460,0.0476)	0.0570 (0.0543,0.0597)	0.0808 (0.0730,0.0893)
70	0.0341 (0.0384,0.0302)	0.0543 (0.0569,0.0517)	0.0668 (0.0677,0.0657)	0.0820 (0.0805,0.0833)	0.0994 (0.0947,0.1040)	0.1395 (0.1263,0.1537)
80	0.0456 (0.0513,0.0404)	0.0723 (0.0756,0.0689)	0.0887 (0.0899,0.0873)	0.1086 (0.1066,0.1104)	0.1312 (0.1251,0.1373)	0.1826 (0.1658,0.2008)

Table S5.4 Absolute cumulative lifetime risks calculated using PRS values obtained from 77 SNPs and HR (with its %95 confidence intervals)

In addition to comparison of the risk with respect to risk groups, we can also examine the the comparison of the risks of individuals as such. For this aim, we compared the individual cumulative lifetime risks using the 3820 SNPs and the 2803 SNPs model. As given in Figure S4, there is a strong correlation between the individual risks calculated using this model.

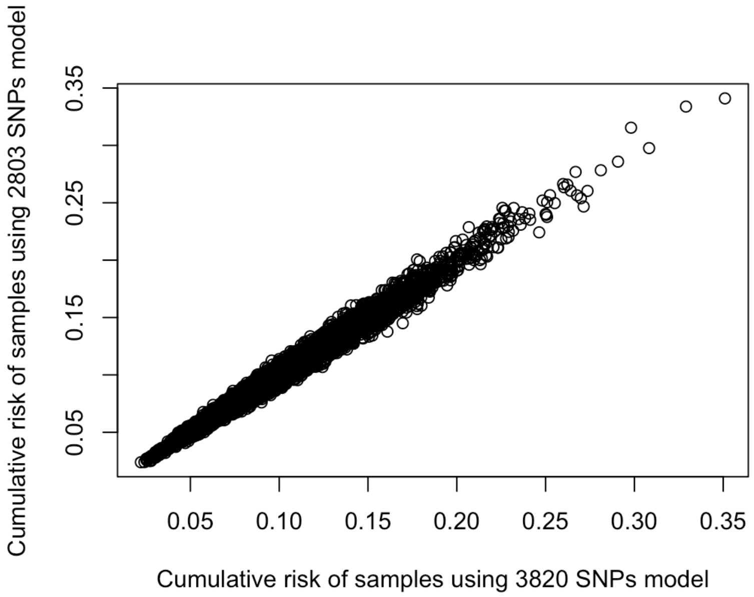

Figure S4. Comparison of the cumulative lifetime risk scores of individuals for the 3820 SNPs and the 2803 SNPs models
